# Prevalence and characteristics of patent foramen ovale in a sample of Egyptian population: a computed tomography study

**DOI:** 10.1186/s43044-024-00504-3

**Published:** 2024-06-10

**Authors:** Ahmed Shehata, Abdulaziz Nasser, Ahmed Mohsen, Amir Anwar Samaan, Amir Mostafa, Mohamed Hassan

**Affiliations:** 1 Cardiology Department, Cairo University, Giza, Egypt; 2 Cardiology Department, AlNas Hospital, Qalyubia, Egypt

**Keywords:** PFO, Prevalence, Interatrial septum abnormalities, CT, Stroke, Syncope

## Abstract

**Background:**

The reported prevalence of patent foramen ovale (PFO) in the general population is variable. It ranges between 8.6 and 42% according to the population studied and the imaging technique used. We aim to prospectively assess the prevalence and characteristics of PFO and interatrial septum (IAS) abnormalities as well as the related clinical manifestations in a sample of Egyptian population.

**Results:**

This study comprised 1000 patients who were referred for CT coronary angiography (CTCA). Mean age was 52.5 ± 10.9 years. The prevalence of PFO among the studied population was 16.3%; closed PFO (grade I) 44.2%, open PFO (grade II) 50.9%, and open PFO with jet (grade III) 4.9%. Anatomical high-risk PFO features—defined as the presence of at least 2 or more of the following (diameter ≥ 2 mm, length ≥ 10 mm, septal aneurysm “ASA”, or redundant septum)—were found in 51.5% of PFOs’ population. Other IAS abnormalities as redundant septum (8.6%), ASA (5.3%), Bachmann’s bundle (4.5%), microaneurysm (2.6%), and atrial septal defect (ASD) (0.4%) were detected. There was a lower rate of coexistence of ASA with PFO (*p* = 0.031). Syncope was significantly higher in patients with PFO compared to those without PFO (6.7% vs. 1.6%, *p* = 0.001). Stroke, transient ischaemic attacks (TIA), and dizziness were similar in both groups. TIA, dizziness, and syncope were significantly higher in patients with IAS abnormalities including PFO compared to those without IAS abnormalities. Syncope was also significantly higher in PFO with high-risk anatomical features compared to those with non-high-risk PFO population (*p* = 0.02).

**Conclusion:**

The prevalence of PFO in our study was approximately 16.3%, almost half of them showed anatomical high-risk features for stroke. Dizziness, syncope and TIA were significantly higher in patients with IAS abnormalities including PFO.

## Background

At birth, foramen ovale valve is functionally closed at birth due to reversal in the pressure gradient between both atria. It eventually closes anatomically permanently in healthy newborns. When it fails to close properly, foramen ovale become patent (patent foramen ovale—PFO) [1]. Universally about a quarter of adults have PFO [2, 3]. A study on Greek population showed a much higher prevalence (42.7% of healthy population, and 49% of patients with cryptogenic stroke) [4]. No data exist regarding the prevalence of PFO in the general population in Egypt.

Several clinical conditions are associated with PFO, including systemic arterial embolism, cryptogenic stroke, migraine headache, decompression illness, and worsening of pre-existent chronic lung disease [5, 6]. In addition, other symptoms could be related to PFO such as dizziness, syncope, chest tightness, and palpitation [7–9].

There are different modalities (such as transesophageal echocardiography “TEE”, or computed tomography “CT”) can help to detect PFO. *CT is a non-invasive and well-validated method for diagnosis of PFO, using TEE as a reference gold standard method *[10, 11]*. CT constitutes an efficient, and practical alternative to echocardiography for detection of PFO, clear visualization of anatomical details of the interatrial tunnel, and detection of other interatrial septal abnormalities (IAS) abnormalities such as atrial septal aneurysms (ASA)* [12, 13].

There is a need to know the prevalence and anatomical characteristics of PFO in our population as well as its associated clinical manifestations. This will allow early intervention and help in improving PFO-related manifestations and preventing further sequelae.

The aim of our work is to assess the prevalence and characteristics of PFO and other IAS abnormalities as well as the related clinical manifestations in a sample of Egyptian population.

## Methods

This was a prospective observational cohort study that enrolled 1000 patients (18 years or older) who were referred for CT coronary angiography in a radiology centre in Cairo-Egypt from March 2023 to October 2023. A written informed consent was obtained from all study participants. Patients who have poor cardiac CT windows, post-cardiac surgery, valvular heart diseases, pulmonary hypertension, and chronic obstructive pulmonary disease were excluded. Patients were thoroughly assessed with special emphasis on the following:

*Demographics:* Age (patients were further divided into three age groups; below 40 years, 41–60 years, above 60 years), and gender.

*Risk factors for cardiovascular diseases:* Diabetes Mellitus (DM): defined by fasting plasma glucose level ≥ 126 mg/dl, HbA1c > 6.5% or on therapy [14]. Smoking status: defined as current cigarette or shisha smokers or those who recently quit smoking [15]. Hypertension (HTN): defined as the use of anti-hypertensive medications or the persistent elevation of blood pressure above 140/90 mmHg on two or more occasions [16]. Hypercholesterolemia: defined as an elevation of plasma total and LDL cholesterol above the target [17]. Family history of premature coronary artery disease (female < 65 years, male < 55 years).

*Clinical manifestations:* History of transient ischaemic attack (TIA) which was defined as a focal neurological deficit which resolved completely within 24 h [18]. History of ischaemic stroke which was defined as sudden neurological deficit that persisted beyond 24 h [18]. The presence of atrial fibrillation (AF) was assessed [19]. Migraine headache which was defined as recurrent episodes of headache most often unilateral with (MA+) or without (MA−) visual or sensory symptoms (aura) [20]. Syncope which was defined as transient loss of consciousness with loss of postural tone that is followed by spontaneous recovery without any interventions [21]. Dizziness which was defined as a disturbed sense of relationship to space [22]. Chest tightness and palpitation were also assessed.

### CT angiography protocol

*Scan Parameters:* All CT angiography examinations were performed using the dual source CT scanner SOMATOM Force 384 (2 × 192) slices (Siemens SOMATOM force, Erlangen, Germany). Scanning parameters were: 192 × 0.25 mm collimation, tube rotation time of 250 ms, tube voltage of 70 kV (increased to 150 kV in obese patients) and current of 292–600 mA. The field of view was 35.4 cm with an image matrix of (512 × 512 pixels) up to (1024 × 1024 pixels). The scanning direction was cranio-caudal.

*Contrast material:* For contrast enhancement, non-ionic contrast medium (Ultravist 370 Schering, Berlin, Germany) was injected through 18-gauge cannula into an upper limb vein with a flow rate of 5–6 mL/s using a programmed dual-head injector pump (MedRad; USA) with dose adjustment according to the patient’s weight. Injection of 40 ml of chasing saline to push the injected contrast material and to wash-out the right side of the heart was done.

*Scan protocol:* The patients were placed in a supine position on the table of the CT machine. All studies were ECG-gated with retrospective reconstructions. Oral β-blockers were administered when the resting heart rate of the patient exceeds 70 beats per minute. In case of AF, the ventricular rate should be properly controlled “not more than 80 beats per minute” with using retrospective ECG gating and current modulation. The field of view was centred two cm to the left of the dorsal spine on the AP scout and at the level of the hilum on the lateral scout. The total effective patient radiation dose at this stage of the scanning was minimized with the use of a relatively low tube current. Bolus tracking technique was used to determine when contrast density is optimal for coronary imaging. Scanning was performed during a single breath hold. Standard axial and reformatted 2D multiplanar images were used for interpretation. Sagittal and axial views through the IAS were specifically evaluated for the presence of each of the CT criteria for PFO outlined below and for the presence of other IAS abnormality such as ASA and redundant atrial septum.

*Image analysis and measurement:* Standard axial, sagittal images were acquired at the best phases in systole and diastole. Multiplanar reformations of the images (short-axis, two-chamber, and four- chamber views) were rendered and evaluated. The length of the free flap valve (FFV) and the distance between the FFV and the septum secundum of the PFO tunnel were measured. FFV length was measured in oblique sagittal images perpendicular to the IAS on four-chamber views as well as in axial view. The flap valve was measured from its free margin [23].

The diameter of the PFO tunnel was measured at midpoint. The maximum length of the contrast material jet was also measured.

**Fig. 1:**
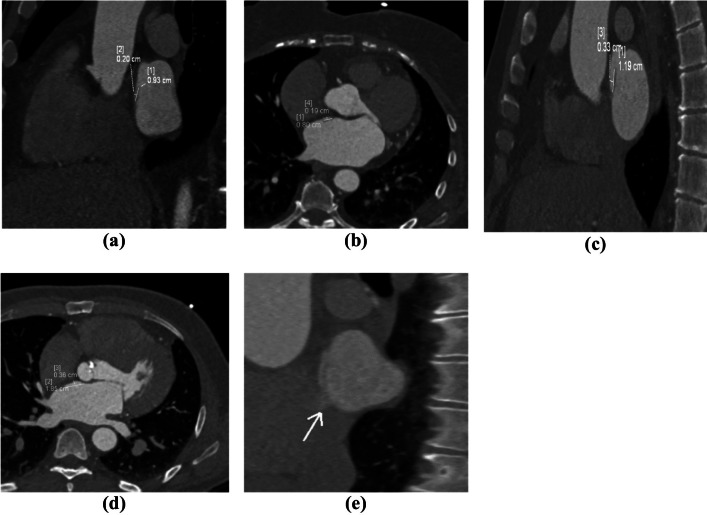
Classification of PFO. (**a, b**) Grade I closed PFO in sagittal oblique and axial views. (**c, d**) Grade II open PFO in sagittal oblique and axial views. (**e**) Grade III open PFO with jet contrast seen in the right atrium (white arrow) in sagittal oblique view.

PFO was then classified according to Williamson et al. classification system [23] into: (1) Grade I (closed flap): a distinct flap at the expected location in the left atrium (LA). (2) Grade II (open flap): a continuous column of contrast material between the septum primum and septum secundum connecting the LA and right atrium (RA). (3) Grade III (open flap with jet): an open flap plus a jet of contrast material from the column into the RA (Fig. 1).

High-risk anatomical PFO features for stroke [24] were defined as the presence of at least 2 or more of the following features: Diameter ≥ 2 mm as measured by the maximum separation between the septum secundum and septum primum, length ≥ 10 mm as measured by the maximum overlap between the septum secundum and septum primum, ASA, and redundant interatrial septum.

**Fig. 2:**
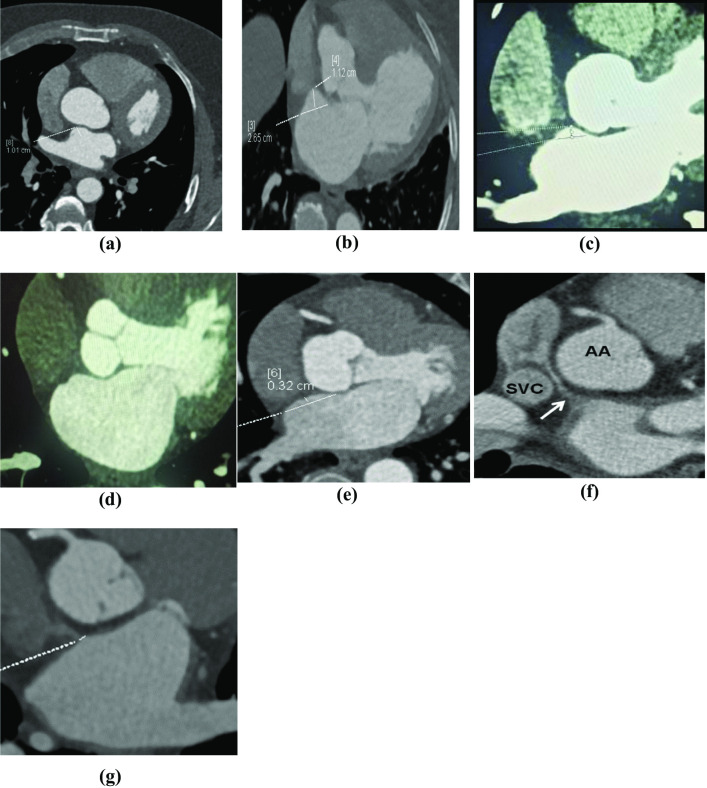
Different IAS abnormalities other than PFO. (**a, b**) Axial view showing ASA with septal excursion ≥ 10 mm from the midline. (**c**) Axial view showing IAS outpouching (microaneurysm) with septal excursion < 10 mm from the midline. (**d, e**) Axial view showing redundant IAS. (**f, g**) Axial view showing Bachmann’s bundle

ASA is defined as redundant and hypermobile portion of IAS that demonstrated ≥ 10 mm excursion from the centre line. IAS microaneurysm is redundant and hypermobile portion of interatrial septum demonstrated < 10 mm excursion from the centre line. Redundant IA in which the IAS deviated from the midline and measured in axial view. (Fig. 2).

Bachmann’s bundle (BB) is a band-like structure of which the site of attachment to the RA or LA was identified. The length of the BB was measured along its anterior margin in axial view (anteroposterior). (Fig. 2).

### Statistical analysis

Statistical analysis was performed using Statistical Package for Social Sciences, version 16 (SPSS 16). Quantitative variables were tested for normality using Shapiro–Wilk test, graphs and histograms. Normally distributed data are presented as mean and standard deviation. Data which are not normally distributed are presented as median (range). Qualitative data are presented as number (percentage). The statistical significance of the difference between normally distributed data were assessed using Student t test. Variables were compared between two unrelated samples using Mann–Whitney U test. Categorical variables were compared using × 2 analysis. Fisher Exact test was used to examine the relationship between two qualitative variables (syncope) when the expected count is less than 5 in more than 20% of cells. A p value is considered significant if < 0.05 at confidence interval (CI) 95%. Binary logistic regression was used to control for smoking as a confounder.

*Assessment of reliability:* CT analysis was performed by a single expert operator. Test–retest reliability was done by repeating the analysis of the data of the first fifty patients after one week. The intra-class correlation coefficient was measured and ranged from 0.88 to 0.98.

## Results

The majority of patients in our study were male (60.5%). The mean age was 52.5 ± 10.9 years. Clinical characteristics are described in Table 1. The most common clinical presentation among the study population was chest tightness (70.5%), followed by palpitation (30.7%) then atrial fibrillation (15.2%), dizziness (14.9%), migraine headache (8.1%), stroke (4.9%), TIA (2.5%) and syncope (2.4%). Almost half (48%) of our study patients had no obstructive coronary artery disease (CAD). There was no significant difference in the clinical presentations between patients with obstructive CAD compared to those with no CAD.

**Table 1:** Demographic, clinical characteristics and presentation of the study population

Clinical characteristics	
Age (years)	52.5 ± 10.9
18–40	134 (13.4)
41–60	599 (59.9)
> 60	267 (26.7)
Gender (Male)	605 (60.5)
DM	388 (38.8)
HTN	559 (55.9)
Smoking	357 (35.7)
Dyslipidemia	620 (62.0)
Family history of CVD	18.9 (18.9)
Stroke	49 (4.9)
TIA	25 (2.5)
AF	152 (15.2)
Migraine headache	81 (8.1)
Dizziness	149 (14.9)
Palpitation	307 (30.7)
Chest tightness	705 (70.5)
Syncope	24 (2.4)

Data are presented as mean ± SD, number (%)

*DM* Diabetes Mellitus, *HTN* Hypertension, *CVD* Cardiovascular Disease, *TIA* Transient ischaemic attack, *AF* atrial fibrillation

*PFO and other IAS abnormalities:* Prevalence of PFO was approximately 16.3% among the study population. Of these patients, 44.2% had a grade I PFO (closed flap), 50.9% had a grade II PFO (open flap), and 4.9% had a grade III PFO (open flap with jet). The median diameter and median length of PFO was 2.3 mm and 10.3 mm, respectively. (Table 2).

**Table 2:** PFO among the study population

Variable	
PFO	163 (16.3)
Grade I (closed flap)	72(44.2)
Grade II (open flap)	83(50.9)
Grade III (open flap with jet)	8 (4.9)
Diameter (mm)	2.3 (0.2–14.8)
Length (mm)	10.3 (1.0–22.1)

Data were presented as *n* (%), median (range)

The median septal deviation from the midline in axial view of the redundant septum was 2.8 mm ranging from (1.1–14.0 mm). Out of 26 patients who had microaneurysm, the mean diameter was 3.8 ± 2.9 mm and the mean length was 6.5 ± 1.9 mm. Regarding 53 patients who had ASA, the mean diameter was 4.4 ± 2.5 mm and the mean length was 14.2 ± 3.4 mm. BB was detected in 45 patients. (Median diameter was 2.2 mm (1.1–7.5 mm), and the median length was 12.3 mm (2.7–18.9 mm).)

Out of four patients who had ASD, the median diameter was 10.5 mm (4.3–22.2 mm), and the median jet length was 12.7 mm (12.1–14.8 mm). Patients with PFO have significantly higher rate of coexistence with ASD (1.8% vs. 1.0%, *p* = 0.015) and lower rate of coexistence with ASA (1.8% versus 6%, *p* = 0.031). Anatomical high-risk features of PFO were present in 84 (51.5%) of those patients with PFO.

There was no statistically significant difference in age or gender between patients with and without PFO. Smoking was significantly higher in patients with PFO (*p* = 0.005); however, other CV risk factors were similar in both groups.

*PFO and clinical manifestations:* Syncope was significantly higher in patients with PFO compared to those without PFO (*p* < 0.001); however, there was no significant difference in the incidence of stroke, TIA, AF, migraine headache, dizziness, palpitation, or chest tightness between patients with and without PFO (Table 3).

**Table 3:** PFO and related clinical manifestations

Clinical manifestation	PFO		*p*
	No (*n* = 837)	Yes (* n* = 163)	
Stroke	41 (4.9)	8(4.9)	0.996
TIA	20 (2.4)	5(3.1)	0.585
AF	125 (14.9)	27 (16.6)	0.596
Migraine headache	63 (7.5)	18 (11.0)	0.132
Dizziness	118 (14.1)	31 (19.0)	0.107
Palpitation	253 (30.2)	54 (33.1)	0.462
Chest tightness	587 (70.1)	118 (72.4)	0.562
Syncope	13 (1.6)	11 (6.7)	**0.001**

Data are presented as no (%)

*AF* atrial fibrillation, *TIA* transient ischaemic attack

*All IAS abnormalities and clinical manifestations:* TIA was significantly higher in patients with any IAS abnormalities including PFO compared to those without IAS abnormalities (*p* = 0.006). Similarly, syncope and dizziness were significantly higher in patients with any IAS abnormalities including PFO compared to those without IAS abnormalities (*p* = 0.014, *p* = 0.038, respectively) (Table 4).

**Table 4:** IAS abnormalities and related clinical manifestations

	No IAS abnormality (* n* = 648)	PFO only (* n* = 141)	PFO or other IAS abnormalities (* n* = 210)	*p*
Stroke	29 (4.5)	7 (5.0)	13(6.2)	0.775
TIA	9 (1.4)	5(3.5)	11 (5.2)	**0.006**
AF	93(14.4)	24(17.0)	35(16.6)	0.595
Migraine headache	49 (7.6)	16 (11.3)	16 (7.6)	0.313
Dizziness	83(12.8)	25(17.7)	41 (19.4)	**0.038**
Palpitation	190 (29.3)	47 (33.3)	70 (33.2)	0.439
Chest tightness	460 (71.0)	102(72.3)	143 (67.8)	0.589
Syncope	9 (1.4)	7(5.0)	8 (3.8)	**0.014**

	IAS abnormalities		
	No IAS abnormality (* n* = 648)	Presence of IAS abnormality	*p*
		(* n* = 352)	
Stroke	29 (4.5)	20 (5.7)	0.399
TIA	9 (1.4)	16 (4.5)	**0.002**
AF	93 (14.4)	59 (16.8)	1.027
Migraine headache	49 (7.6)	23 (9.1)	0.717
Dizziness	83 (12.8)	66 (18.8)	**0.012**
Palpitation	190 (29.3)	117 (33.2)	0.200
Chest tightness	460 (71.0)	245 (69.6)	0.646
Syncope	9 (1.4)	15 (4.3)	**0.005**

*P* value significant < 0.05

Smoking was coincidently found to be significantly higher in higher in patients with PFO compared to those without PFO (45.4% vs. 8%, *p* = 0.005); however, other CV risk factors “DM, HTN, dyslipidemia, and family history” were similar between the two groups. Therefore, binary logistic regression analyses were conducted for syncope, dizziness and TIA as outcome variables, while PFO as an independent variable with the addition of smoking status in the regression model as a potential confounder. There was no significant association between smoking and syncope (*p* = 0.062), dizziness (*p* = 0.97), or TIA (*p* = 0.38).

*PFO with high-risk anatomical features:* Syncope was significantly higher in patients with PFO with high-risk anatomical features (*p* = 0.022); however, there was no significant difference in the incidence of stroke, TIA, AF, migraine headache, dizziness, palpitation, or chest tightness between patients with and without PFO.

There was a significant association between syncope and coexistence of PFO with redundant septum (*p* = 0.027). Meanwhile, there was no significant differences between stroke, TIA, AF, migraine headache, dizziness, palpitation and chest tightness in case of coexistence of these structures.

## Discussion

The prevalence of PFO in the general population is variable, ranging between 8.6 and 42.7% (Table 5). The actual prevalence of PFO in Egypt is not known yet. To the best of our knowledge, this is the first study to assess the prevalence of PFO in the Egyptian population. Diagnosis and recognition of PFO would facilitate early intervention, help in improving PFO- and IAS-related manifestations such as stroke, TIA, migraine headache, dizziness and syncope.

**Table 5:** Prevalence and characteristics of PFO in different population studies

	Our study (* n* = 1000) (Egypt,2023)	Kara et al (* n* = 782) (Turkey, 2015)	Purvis et al (* n* = 261) (UK, 2009/2010)	Saremi et al (* n* = 264) (USA, 2008)	Koutroulou et al (* n* = 124) (Greece, 2016–2019)	Kosehan et al (* n* = 1004) (Turkey 2008–2010)	Fisher et al (* n* = 1000) (USA, 1995)
Imaging technique	CT	CT	CT	CT	TEE	CT	TEE
PFO	163 (16.3)	118 (15)	59 (22.6)	101 (38.3)	53 (42.7)	86 (8.6)	92 (9.2)
Grade I (closed flap)	72 (44.2)	30 (25.4)	30 (51)	62 (61.4)	–	–	–
Grade II (open flap)	83 (50.9)	14 (11.9)	17 (29)	35 (34.1)	–	–	–
Grade III (open flap with jet)	8 (4.9)	74 (62.7)	12 (20)	4 (4.5)	–	–	–

Data are presented as no (%)

*CT* computed tomography, *TEE* transesophageal echocardiography

Almost 16.3% of the study population had PFO, 44.2% were grade I, 50.9% were grade II and only 4.9% patients were grade III. In concordance with our results, Kara et al. [13] reported a PFO prevalence of 15% in 782 Turkish patients underwent CT, where 62.7% had grade III, 11.9% had grade II, and 25.4% had grade I PFO. Furthermore, Purvis et al. [25] studied 261 patients from UK by CT, and demonstrated PFO in 22.6% of patients: grade I (51%), grade II (29%), and grade III (20%).

In contrast, other studies showed a lower PFO prevalence results that could be attributed to difference in the sample size, geographical distribution, methodology, as well as the modality of imaging (TEE vs. CT) used. Kosehan et al. [26] have evaluated a total of 1004 adult patients from Turkey retrospectively for interatrial shunting during CT angiography; 8.6% were diagnosed to have PFO, and similarly Fisher et al. [27] reviewed 1000 consecutive TEEs performed with contrast for patients from USA, and reported PFO in 9.2% of patients.

On the other hand, some studies reported a significantly higher prevalence. Saremi et al. [28] reviewed CCTA of 264 patients from USA and PFO was seen in 38.3% (grade I = 61.4%; grade II = 34.1%; and grade III = 4.5%). Similarly, Koutroulou et al. study [4] revealed a prevalence of PFO around 42.7% in Greece population.

In the current study, there was no statistically significant difference in terms of age, age groups, gender in patients with or without PFO. PFO was predominantly present among the age group 41–60 years (62%), age group > 60 years (22.7%), and less commonly found among age group < 40 years (15.3%).

## PFO and cerebrovascular events (CVE)

The precise mechanism by which PFO causes a stroke is uncertain. Paradoxical embolism is the most acceptable hypothesis. Other hypothesis supports in situ clot formation and this hypothesis empowered by the presence of long tunnelled PFO, or concomitant presence of ASA [29].

In the current study, TIA was significantly higher in patients with any IAS abnormalities including PFO compared to those without IAS abnormalities (4.5% vs. 1.4%, *p* = 0.006) with no significant difference in the incidence of stroke between the two groups.

De Castro et al. [30] have identified PFO, by contrast TEE, in 29% of patients with acute ischaemic stroke or TIA. One fourth of these patients had cryptogenic stroke. The risk of CVE was higher in patients with PFO and concomitant septal aneurysm (OR 4.00; 95% CI 2.63–6.09; *p* < 0.001) and with large right-to-left shunt PFO [31].

Trans-catheter PFO closure has been reported to be reduce the risk of recurrent stroke and/or TIA when compared to medical treatment, (HR = 0.59, *p* = 0.04) [32]. A recent meta-analysis showed that PFO closure was associated with a significant reduction in stroke in patients with large PFO shunts; however, there was no significant reduction in stroke in patients with a small shunt [33].

The lower results of stroke and TIA in our study may be attributed to the sample size, different patients’ risk factor profile, and patient selection methods since the previously mentioned studies were done among patients who had already CVE.

## PFO and syncope

Syncope is a common clinical presentation which is associated with poor quality of life, significant disability and death in many cases. The aetiology of such condition still cannot be identified in approximately 50% of patients after a comprehensive cardiovascular and neurological evaluation. Therefore, identifying the predisposing factors in patients with unexplained syncope is crucial for effective preventive measures. In the current study, syncope was significantly higher in patients with PFO, and those with any IAS abnormalities compared to those without PFO or any IAS abnormalities. Right to left shunt (RLS) of venous blood, emboli, and vasoactive substances is suspected to be the underlying mechanism which can precipitate syncope during exercise or changes in abdominal pressure, but limited evidence is available regarding this mechanism [34, 35].

Liu et al., demonstrated a higher incidence of RLS among patients presented with syncope compared to an age and gender matched control group. The association between unexplained syncope and RLS persisted after adjusting for small vessels disease burden [36]. Interestingly, PFO closure may prevent the recurrence of syncope, and the prevalence of recurrent syncope was much lower in patients who underwent PFO closure than that of the non-closure group (11.0% vs. 35%, *p* = 0.018) [37]. Nevertheless, the evidence that percutaneous PFO closure can effectively prevent the recurrence of unexplained syncope is still limited.

## PFO and dizziness

The precise mechanism of dizziness in PFO is still controversial. However, the most acceptable theory is that micro emboli and vasoactive substances bypass the pulmonary circulation through PFO and directly enter the brain, acting on the vestibular system and causing dizziness. Furthermore, dizziness may be related to local vestibular cortex suppression caused by a microbubble embolism that does not cause CVE [38].

In our study, dizziness was reported by 15% of the patients and it was significantly higher among patients with any IAS abnormalities including PFO compared to those without IAS abnormalities (18.8% vs. 12.8%, *p* = 0.038). Dizziness was numerically higher in patients with PFO compared to those without PFO but did not reach statistical significance (19% vs. 14.1%, *p* = 0.1). By review of literature, there were heterogeneities of results of PFO related dizziness. Zhang et al. [33] has demonstrated that 63% of PFO patients were accompanied with dizziness. Moreover, in a large single-centre, prospective, controlled study by Cao et al. [38], the prevalence of PFO in patients with unexplained dizziness was 64.7% compared to 27.4% in those with unexplained dizziness. (*p* < 0.001).

## PFO and AF

AF was present in 15.2% of our study patients, which is higher than reported in other studies. Meissner et al. [39] showed that the prevalence of AF among studied population was only 7.0%. This may be explained by higher prevalence of CAD in our study patients since there is a strong correlation between the two diseases. In the current study, there was no significant difference in the incidence of AF between patients with PFO or any IAS abnormalities compared to those without IAS abnormalities. In a recent systematic review and meta-analysis, a lower incidence of AF in patients with PFO compared with those without (RR 0.52, *p* < 0.001) was reported [40]. In addition, Ahmed et al. [41] showed a lower incidence of AF among PFO patients, which significantly increased after PFO device closure (RR 4.68, *p* < 0.001) [42].

## PFO and migraine headache

Migraine headache was detected in 8.1% of our studied population. Migraine headache was numerically higher in patients with PFO compared to those without PFO but did not reach statistical significance. The association between PFO and migraine has been previously documented in several studies. Caputi et al. [43] investigated the presence of RLS—using contrast enhanced transcranial Doppler- in 159 patients complaining of migraine with aura (MA +). RLS was detected in almost half of the patients and 7.5% of them experienced a typical attack of MA + following the test.

Zhang et al. [35] reported a prevalence of migraine in 34% of a group of PFO patients. In addition, Anzola et al. [44] showed a prevalence of PFO of 48% in patients with MA + , 23% in patients with MA-, and 20% in age matched non-migraine subjects. Two pathophysiological mechanisms have been suggested to explain the association between migraine and PFO: (1) vasoactive substances bypassing metabolism in the lungs via the PFO and acting on the brain directly causing migraine. (2) Tiny brain infarcts caused by paradoxical emboli initiating cortical spreading depression leading to recurrent migraine headache.

## Anatomical high-risk features of PFO

*Diameter and length of PFO:* In our study, the median PFO diameter was 2.30 (0.2–14.8) mm and the median length was 10.30 (1.1–22.1) mm. There was no significant difference in diameter and length of PFO between patients with CVE and those without CVE. The relation between PFO morphometries and associated CVE in the literature are heterogeneous (Table 6).

**Table 6:** Different PFO morphometries in different studies

PFO Morphometrics	Our study 2023	Goel et al. [45]	Komar et al. [46]	Kara et al [13]	Schuchlenz et al. [47]	Holda et al. [31]
Asymptomatic (no CVE) PFO						
Diameter (mm)	2.4 (1.1–3.5)	2.9 ± 1.4	1.3 ± 1.3	2.0 ± 1.0	2.0 ± 1.0	1.8 ± 1.4
Length (mm)	10.1(1.1–22.1)	12.0 ± 6.0	12.0 ± 5.5	12.1 ± 1.0	–	10.4 ± 9.1
Symptomatic (CVE) PFO						
Diameter (mm)	2.3 (0.2–14.8)	3.9 ± 1.6	3.9 ± 1.4	–	4.0 ± 2.0	2.4 ± 1.5
Length (mm)	11.4 (7.9–15.2)	14.0 ± 6.0	14.0 ± 6.0	–	–	10.8 ± 8.6

Data are presented as median (range), no (%)

Schuchlenz et al. [47] reported that the mean PFO diameter (by TEE) was significantly larger in patients with CVE than in control subjects (*p* < 0.0001). A PFO greater than 4 mm was associated with a significantly higher risk of TIA, ischaemic strokes (OR = 27; 95%, *p* = 0.0002). The size of PFO has been demonstrated to be an independent risk factor for stroke occurrence and recurrence (OR of 2.54 when the size is ≥ 2 mm) [48].

*ASA:* ASA was detected in 5.3% of our study patients and coexisted with PFO in only 1.8% of patients. There was lower rate of association between ASA and PFO (*p* = 0.031). In the present study, there was no significant difference between coexistence of PFO with ASA and presence of CVE. In concordance with our results, Di Tullio et al. [34] detected ASA in 2.5% of study patients, and both PFO and ASA were found in only 1.7%. In that study, the coexistence of PFO and ASA was not found to increase the risk of stroke. In contrast, Cabanes et al. [49] showed that the odds of stroke in a patient with coexisting ASA and PFO were 33.3 times. Similarly, Mas et al. [50] showed that the OR for stroke was 4.96 when PFO and ASA coexisted, compared with 1.83 for PFO or 2.35 for ASA in isolation.

*Redundant septum (Floppy septum):* Redundant septum was detected 8.6% of our study patients and in 6.7% of patients it coexisted with PFO. There was no significant association between redundant septum and PFO (*p* = 0.357), and there was no significant difference between coexistence of redundant septum with PFO and presence of CVE. On the contrary to our results, Lee et al. [51] demonstrated that ASA or hypermobility of the atrial septum (detected by TEE) were independent predictors of stroke recurrence (hazard ratio 6.04, *p* = 0.003).

The study has the following limitations: (1) All patients were taken from a single centre and specific geographical distribution. (2) The definitive aetiology of CVE in our study could not be determined (whether cryptogenic or due to AF, or the presence of a definite or possible arterial thrombosis or small vessel disease) since we include all patients referred for CTCA meeting the inclusion criteria.

## Conclusions

The prevalence of PFO in this study was approximately 16.3%, almost half of them showed anatomical high-risk features for stroke. Dizziness, syncope and TIA were significantly higher in patients with IAS abnormalities including PFO.

## Data Availability

The datasets used and/or analysed during the current study are available from the corresponding author on reasonable request.

## Abbreviations

PFO: Patent foramen ovale
IAS: Interatrial septum
TIA: Transient ischaemic attack
AF: Atrial fibrillation
CT: Computed tomography
ASA: Atrial sepal aneurysm
ASD: Atrial septal defect
TEE: Transesophageal echocardiography
LA: Left atrium
RA: Right atrium
BB: Bachmann’s bundle
CCTA: CT coronary angiography
CVE: Cerebrovascular events
CAD: Coronary artery disease
